# Complimentary

**Published:** 1888-11

**Authors:** 


### COMPLIMENTARY.

We desire to call attention to the report of the American and Southern Dental Societies meeting, which is begun in this number by Mrs. M. W. J. While it is only an abstract, and such, by the way, is all we ever intend to print of reports that belong to other journals, yet Mrs. W. has caught the salient points so accurately that no one, even of those who were in attendance, will miss what has been omitted. We have never seen any report that was freer from error.

We can well understand, how, by a system of short-hand, a detailed report can be obtained, providedthe reporter is conversant with technical terms.

It is remarkable how a person not acquainted with short-hand, could so enter into the spirit of the meeting as to give such an accurate a report.

We gladly give this note of commendation to Mrs. W., because in her case it is deserved; and because of the fact that the general impression is extant that women cannot, or at least do not, make good reporters. Here at least is a notable exception to the rule, if such it be. What one woman has done others can do, and we believe that the reason that most women have not entered this light and most suitable field of employment is because of lack of opportunity and special training rather than any unfitness of mind or body for the occupation.

